# Effect of Gd^3+^, La^3+^, Lu^3+^ Co-Doping on the Morphology and Luminescent Properties of NaYF_4_:Sm^3+^ Phosphors

**DOI:** 10.3390/ma16062157

**Published:** 2023-03-07

**Authors:** Viktor G. Nosov, Anna A. Betina, Tatyana S. Bulatova, Polina B. Guseva, Ilya E. Kolesnikov, Sergey N. Orlov, Maxim S. Panov, Mikhail N. Ryazantsev, Nikita A. Bogachev, Mikhail Yu Skripkin, Andrey S. Mereshchenko

**Affiliations:** 1 Saint-Petersburg State University, 7/9 Universitetskaya Emb., 199034 St. Petersburg, Russia; 2 Federal State Unitary Enterprise “Alexandrov Research Institute of Technology”, 72 Koporskoe Shosse, 188540 Sosnovy Bor, Russia; 3 Institute of Nuclear Industry, Peter the Great St. Petersburg Polytechnic University (SPbSU), 29, Polytechnicheskaya Street, 195251 St. Petersburg, Russia; 4 Center for Biophysical Studies, Saint Petersburg State Chemical Pharmaceutical University, 14 Professor Popov Str., Lit. A, 197022 St. Petersburg, Russia; 5 Nanotechnology Research and Education Centre RAS, Saint Petersburg Academic University, 8/3 Khlopina Street, 194021 St. Petersburg, Russia

**Keywords:** luminescence, microcrystals, nanocrystals, hydrothermal synthesis, rare earth, samarium, co-doping

## Abstract

The series of luminescent NaYF_4_:Sm^3+^ nano- and microcrystalline materials co-doped by La^3+^, Gd^3+^, and Lu^3+^ ions were synthesized by hydrothermal method using rare earth chlorides as the precursors and citric acid as a stabilizing agent. The phase composition of synthesized compounds was studied by PXRD. All synthesized materials except ones with high La^3+^ content (where LaF_3_ is formed) have a β-NaYF_4_ crystalline phase. SEM images demonstrate that all particles have shape of hexagonal prisms. The type and content of doping REE significantly effect on the particle size. Upon 400 nm excitation, phosphors exhibit distinct emission peaks in visible part of the spectrum attributed to ^4^G_5/2_→^6^H_J_ transitions (J = 5/2–11/2) of Sm^3+^ ion. Increasing the samarium (III) content results in concentration quenching by dipole–dipole interactions, the optimum Sm^3+^concentration is found to be of 2%. Co-doping by non-luminescent La^3+^, Gd^3+^ and Lu^3+^ ions leads to an increase in emission intensity. This effect was explained from the Sm^3+^ local symmetry point of view.

## 1. Introduction

Lanthanide-doped inorganic materials have been attracting much attention from scientists for several decades. These materials have promising applications in medicine and technology as materials for optical devices, sensing, tumor therapy, bioimaging, drug delivery, anti-counterfeiting, optical thermometry, etc. [1,2,3,4,5,6,7,8,9].

The optical properties of these materials depend on the particles’ size and morphology, crystal symmetry, type, and concentration of rare earth ions in the host matrix [10,11,12,13,14,15,16]. Sodium yttrium fluoride is one of the best host matrices for luminescent rare earth-doped inorganic materials because this matrix has only low-frequency vibrational modes, and therefore does not quench the luminescence. In addition, NaYF_4_ possesses chemical inertness, low toxicity, and the possibility to combine magnetic, optical, and radioactive properties of lanthanide ions that opens the way to prepare new theranostic agents for non-invasive therapy [17,18,19,20,21,22]. As a co-dopant, lanthanide ions play several key roles in photoluminescent materials: they may absorb light as sensitizers or emit photons as luminescence activators as well as transfer energy from the sensitizer to activator [13,14,23,24,25]. At the same time, the addition of non-luminescent dopants (e.g., alkali, alkali earth, some *p*-, *d*- and *f*-metal ions) in host matrix doped with luminescent ions is known to enhance the luminescence intensity [11,26,27,28]. This effect is assumed to be caused by several factors: structural changes in the crystal lattice upon doping (e.g., formation of ionic vacancies) and modification of the crystal field surrounding Ln^3+^ activators [28,29,30]. Yet, generally, it is still early to believe that the mechanism of the co-doping effect on luminescence is fully explained because there is no model to predict the impact of any dopant ions on the optical properties of such doped materials. We presumed that this is caused by the deficiency of studies. For example, to the best of our knowledge, the non-luminescent dopants are mainly chosen from non-lanthanide elements. This approach neglects the fundamentally interesting details of the mutual effect of ions on similar electronic structures. Previously we have reported the particle size and shape dependence on the nature of the doping lanthanide (III) ions NaYF_4_:Ln^3+^ series and described the correlation between the obtained nanoparticle morphologies and the type and content of doping ions [10]. We found that the average diameter of particles reaches the least value for Sm^3+^, Eu^3+^, and Gd^3+^ doped materials. We have studied NaYF_4_:Eu^3+^ particles co-doped with Gd^3+^ ions [11] and revealed that Gd^3+^ doping results in particle size reduction as well as the increase in emission intensity and ^5^D_0_ lifetime of europium (III). We have obtained a similar effect of simultaneous size reduction and luminescence intensity enhancement for gadolinium ion-doped materials for NaYF_4_:Yb^3+^, Tm^3+^/Er^3+^ up-conversion microcrystalline materials [16]. Further investigations of up-conversion materials based on NaYF_4_ doped with erbium, ytterbium and co-doped with lutetium ions showed that the addition of optical inactive Lu^3+^ results in both increasing particles size and luminescence intensity [31]. In order to find out whether the luminescence intensity enhancement is the common trend upon doping with gadolinium or other non-luminescent lanthanide ions, we intended to study samarium-containing down-conversion phosphors in the current work.

Samarium compounds are of interest in medicine and the production of functional nanoparticles. For example, the decay energy of the samarium ^153^Sm nuclide allows using this isotope for cancer therapy and SPECT imaging [32,33]. Sm^3+^ ions are also known to be used as a part of optically active materials because of their orange luminescence, originating from the ^4^G_5/2_ → ^6^H_J/2_ (J = 5, 7, and 9) transitions [14,34,35,36,37]. Nevertheless, the works devoted to the co-dopant effect on samarium-doped compounds as a way to control the luminescence properties of these materials are limited, and this effect should be studied in detail.

In this present study, we reported the effect of rare earth doping concentration on the morphology, structure, and luminescence properties of the series of NaYF_4_ compounds doped with Sm^3+^ and co-doped with non-luminescent La^3+^, Gd^3+^, and Lu^3+^ ions and proposed the theoretical explanations of such effects.

## 2. Materials and Methods

Anhydrous chlorides of the rare earth elements (YCl_3_, SmCl_3_, LaCl_3_, GdCl_3_, LuCl_3_, 99.999%) were purchased from Chemcraft (Kaliningrad, Russia), KBr, NaOH, NH_4_F, citric acid, and ethanol were purchased from Sigma-Aldrich Pty Ltd. (Darmstadt, Germany), and used without additional purification.

Microcrystalline β-NaYF_4_ samples co-doped with Sm^3+^, La^3+^, Gd^3+^, and Lu^3+^ were synthesized using the hydrothermal method using citric acid as a stabilizing agent, described previously [11,16]. Rare earth chlorides taken in stoichiometric amounts (total amount of rare earth chlorides was 0.75 mmol) with 3 mmol of citric acid were dissolved in distilled water to obtain 5 mL solution in total. Then, 2.5 mL of an aqueous solution containing 9 mmol of NaOH was added to the reaction mixture. After vigorous stirring for 30 min, 8 mL of aqueous solution containing 11 mmol of NaOH and 11 mmol of NH_4_F was added into the above solution. The solution was maintained after vigorous stirring for 30 min at room temperature before being transferred to a Teflon-lined autoclave with an internal volume of 20 mL and heated for 17h at the temperature of 180 °C. After that, the precipitate was separated from the reaction mixture by centrifugation, washed with ethanol and deionized water, and dried at 60 °C for 24 h. The desired microcrystalline materials were obtained in the form of white powders.

In this work, we synthesized and studied four series of luminescent powders: NaY_1-x_Sm_x_F_4_ (x = 0–0.4) and NaY_0.98−y_Sm_0.2_Ln_y_F_4_ (Ln = La, Gd, Lu; y = 0–0.6). Among NaY_1−x_Sm_x_F_4_ series, materials containing 2% (x = 0.02) of Sm^3+^ demonstrated the highest luminescence intensity (discussed below in the Results and Discussion section). Therefore, to follow the effect of Ln^3+^ (Ln = La, Gd, Lu) co-doping on the luminescence properties, we kept the concentration of Sm^3+^ equal to 2% in the NaY_0.98−y_Sm_0.2_Ln_y_F_4_ series. The relative content of the rare earth elements in the synthesized compounds was confirmed by energy-dispersive X-ray spectroscopy. The particles’ morphology was characterized using scanning electron microscopy (SEM) on a Zeiss Merlin electron microscope (Zeiss, Oberkochen, Germany) using an energy-dispersive X-ray spectroscopy (EDX) module (Oxford Instruments INCAx-act, Oxford, UK). powder X-ray diffraction (PXRD) measurements were performed on a D2 Phaser (Bruker, Billerica, MA, USA) X-ray diffractometer using Cu Ka radiation (λ = 1.54056 Å). To carry out quantitative photoluminescence studies, the synthesized samples (20 mg) and potassium bromide (300 mg) were pressed into pellets (diameter 13 mm). The luminescence spectra were recorded on Fluorolog-3 fluorescence spectrometer (Horiba Jobin Yvon, Kyoto, Japan). Lifetime measurements were performed using the same spectrometer using a pulsed Xe lamp (pulse duration 3 µs).

## 3. Results and Discussion

### 3.1. Crystal Structure

**Figure 1:**
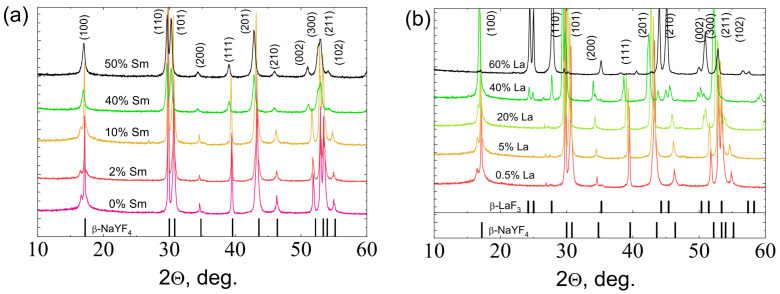

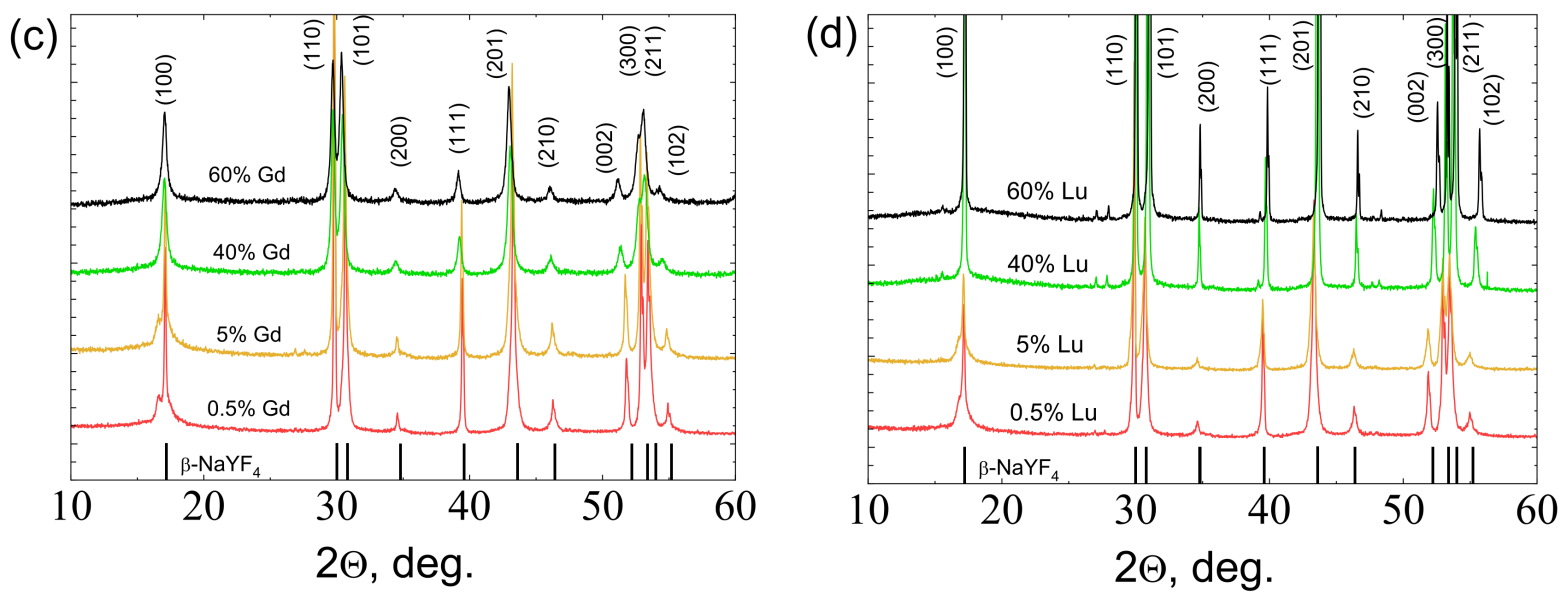
PXRD patterns of (**a**) NaY_1−x_Sm_x_F_4_, (**b**) NaY_0.98−x_Sm_0.02_La_x_F_4_, (**c**) NaY_0.98−x_Sm_0.02_Gd_x_F_4_, and (**d**) NaY_0.98−x_Sm_0.02_Lu_x_F_4_.

The powder X-ray diffraction (PXRD) patterns are shown in Figure 1a–d). Analysis of PXRD patterns demonstrates that all synthesized materials of three series (NaY_1−x_Sm_x_F_4_, NaY_0.98−x_Sm_0.02_Gd_x_F_4_ and NaY_0.98−x_Sm_0.02_Lu_x_F_4_) have the same crystalline phase, which corresponds to the hexagonal β-NaYF_4_ (JCPDS No. 16-0334). Additional diffraction peaks corresponding to the impurities are not observed. In opposition to the abovementioned series, we have found that substitution of yttrium by the lanthanum ions in NaY_0.98−x_Sm_0.2_La_x_F_4_ series results in the formation of either β-NaYF_4_ or LaF_3_ (JCPDS No. 32-0483) crystalline phases depending on the lanthanum content. Thus, at the lanthanum content less 20 at.% and less, only β-NaYF_4_ crystalline phase is formed similarly to other series. At the lanthanum content of 40 at.%, β-NaYF_4_ or LaF_3_ phases coexist. At the content of lanthanum of the 60 at.%, compounds precipitate exclusively in a form of LaF_3_ phase.

**Figure 2:**
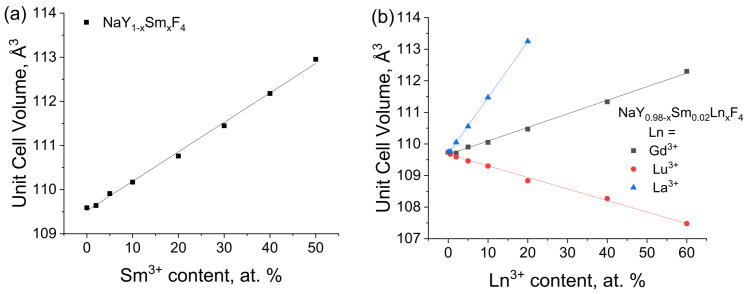
The dependence of unit cell volumes of NaY_1−x_Sm_x_F_4_ on the Sm^3+^ content (**a**) and NaY_0.98−x_Sm_0.02_Ln_x_F_4_ (Ln = La, Gd, Lu) samples on the Ln^3+^ content (**b**).

Unit cell parameters were refined using UnitCell software [38]. This program can retrieve unit cell parameters from diffraction data using a method of least squares from the positions of the indexed diffraction maxima of the PXRD patterns (Pawley method [39]). The uncertainties of unit cell parameters are shown in parenthesis in Tables S1–S4, Supplementary Materials. The dependence of refined unit cell volumes on the sample composition is shown in Figure 2. Unit cell volume linearly depends on dopant concentration, therefore, Vegard’s law [40] obeys the studied systems; hence, Ln^3+^ (Ln = Sm, Gd, Lu, La) ions isomorphically substitutes Y^3+^ ions in the β-NaYF_4_ structure. For compounds NaY_1−x_Sm_x_F_4_, the increase in Sm^3+^ content leads to unit cell volumes increase due to a higher ionic radius of Sm^3+^ ions (1.132 Å, the coordination number is nine) than the ionic radius of Y^3+^ ions (1.075 Å) [41]. Similarly, the doping of NaY_0.98_Sm_0.2_F_4_ by lanthanide (III) ions with higher ionic radius than Y^3+^ ions (Gd^3+^: 1.107 Å; La^3+^: 1.216 Å) results in increasing the unit cell volumes. Moreover, for the NaY_0.98−x_Sm_0.02_La_x_F_4_ series, unit cell volume increases significantly faster than for the NaY_0.98−x_Sm_0.02_Gd_x_F_4_ one because La^3+^ ions have a larger ionic radius than Gd^3+^. Meanwhile, the unit cell volumes for NaY_0.98−x_Sm_0.02_Lu_x_F_4_ series decrease upon lutetium concentration rise, which can be similarly explained by the lower ionic radius of Lu^3+^ ions (1.032 Å) than the ionic radius of Y^3+^ ions.

### 3.2. Morphology

**Figure 3:**
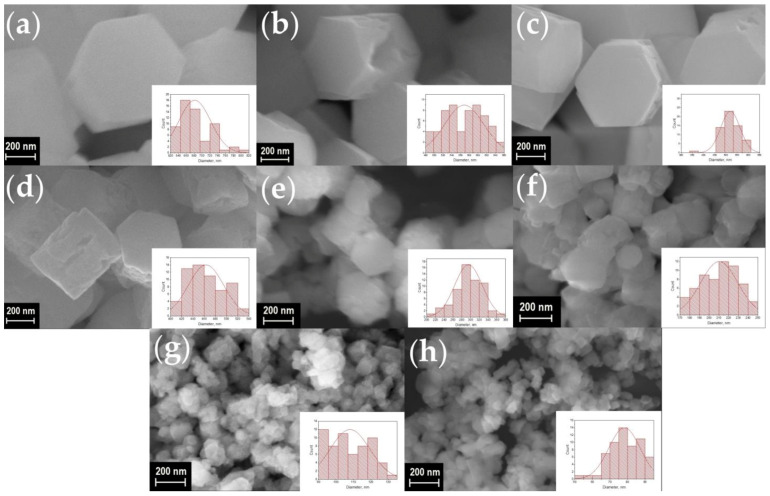
SEM images of the samples NaY_1−x_Sm_x_F_4_ (**a**–**h**): x = 0, 2, 5, 10, 20, 30, 40, and 50 at.% of Sm^3+^. Particle size distribution of the samples is shown in the insets. The average diameter of particles is equal to about 682 ± 41, 568 ± 44, 520 ± 43, 463 ± 31, 295 ± 32, 210 ± 19, 108 ± 11, and 78 ± 9 nm for the Sm^3+^ concentration of 0, 2, 5, 10, 20, 30, 40, and 50 at.%, respectively.

**Figure 4:**
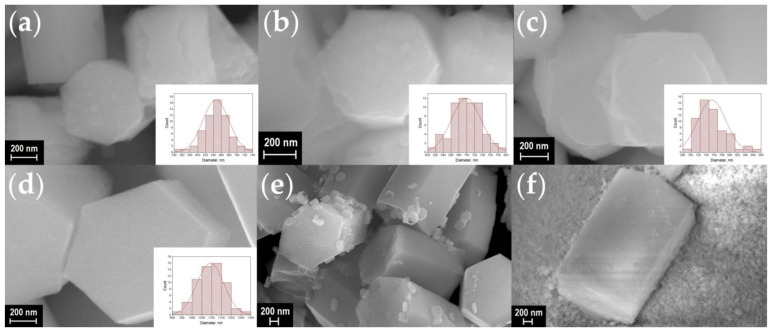
SEM images of the samples NaY_0.98−x_Sm_0.02_La_x_F_4_ (**a**–**f**): x = 2, 5, 10, 20, 40, and 60 at.% La. Particle size distribution of the samples is shown in the insets. The average diameter of particles is equal to about 646 ± 33, 698 ± 38, 754 ± 36, 1094 ± 69, 1517 ± 64 (254 ± 16 for small particles) and 1916 ± 132 (102 ± 9 for small particles) nm for the La^3+^ concentration of 2, 5, 10, 20, 40, and 60 at.%, respectively.

**Figure 5:**
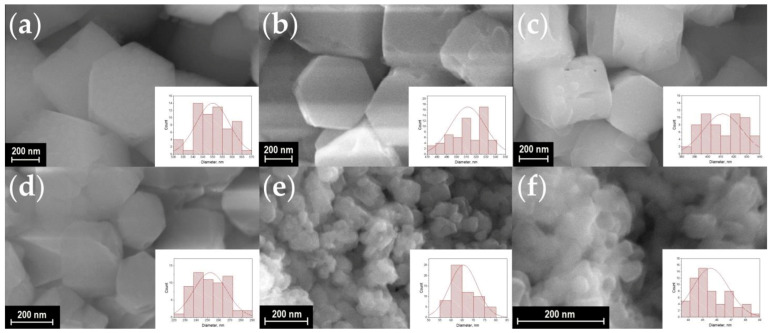
SEM images of the samples NaY_0.98−x_Sm_0.02_Gd_x_F_4_ (**a**–**f**): x = 2, 5, 10, 20, 40, and 60 at.% Gd, respectively. Particle size distribution of the samples is shown in the insets. The average diameter of particles is equal to about 550 ± 9, 511 ± 18, 412 ± 15, 252 ± 15, 66 ± 6, and 46 ± 2 nm for the Gd^3+^ concentration of 2, 5, 10, 20, 40, and 60 at.%, respectively.

**Figure 6:**
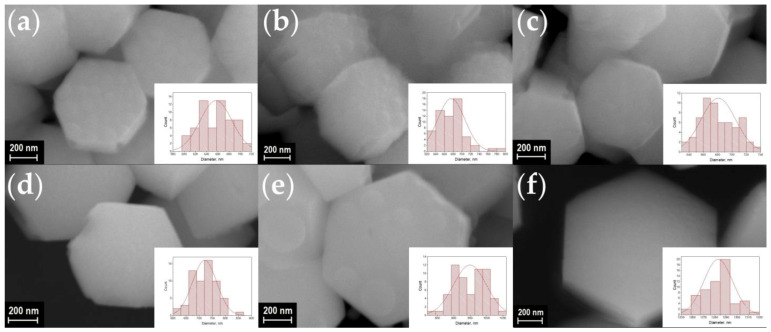
SEM images of the samples NaY_0.98−x_Sm_0.02_Lu_x_F_4_ (**a**–**f**): x = 2, 5, 10, 20, 40, and 60 at.% Lu, respectively. Particle size distribution of the samples is shown in the insets. The average diameter of particles is equal to about, 657 ± 29, 676 ± 31, 681 ± 24, 721 ± 46, 949 ± 50, and 1283 ± 13 nm for the Lu^3+^ concentration of 2, 5, 10, 20, 40, and 60 at.%, respectively.

**Figure 7:**
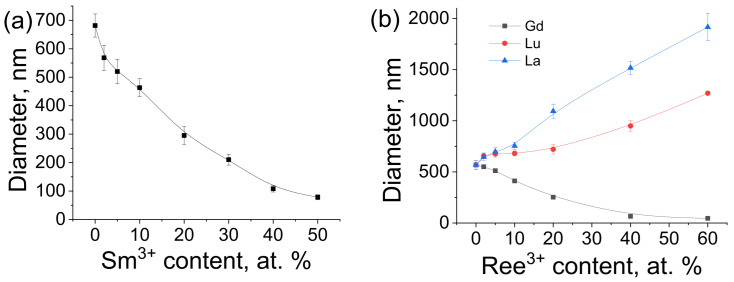
The effect of dopant nature and concentration on NaY_1−x_Sm_x_F_4_ (**a**) NaY_0.98−x_Sm_0.02_Ln_x_F_4_ (**b**) particle size.

A scanning electron microscope (SEM) was used to observe the shape and size of the particles in synthesized materials. SEM images of the synthesized materials are shown in Figure 3, Figure 4, Figure 5 and Figure 6. The particles have the shape of hexagonal prisms. The particle diameter was obtained from SEM images, the particle size distribution is shown in the inserts of Figure 3, Figure 4, Figure 5 and Figure 6. The average diameter of the particle was calculated from this distribution and is given in the legends in Figure 3, Figure 4, Figure 5 and Figure 6. The particle size strongly depends on the sample composition ranging from 46 to 1916 nm. In the NaY_1−x_Sm_x_F_4_ series, the size reduction is observed upon increasing the samarium content, Figure 3 and Figure 7. Thus, the NaYF_4_ particles have an average size of 682 ± 41 nm, whereas NaY_0.5_Sm_0.5_F_4_ particles are significantly smaller, 78 ± 9 nm. In the NaY_0.98−x_Sm_0.02_Ln_x_F_4_ (Ln = La, Gd, Lu) series (Figure 3b, Figure 4, Figure 5 and Figure 6), the substitution of the yttrium ion by the lanthanum and lutetium ions results in particle size increase, whereas particle size reduction is observed upon gadolinium doping, Figure 7. This observation can be explained by the mechanism of crystal growth [10]. We assume that the particle size is determined by nucleation and crystal growth rates. If the nucleation rate is larger than the crystal growth rate, small single crystals are formed. In the opposite case, when nucleation is slow, but crystal growth is fast, large single crystals are formed. The crystal growth rate is significantly affected by the Cit^3−^ and Na^+^ adsorption on (1010) and (0001) facets, respectively [17,42]: adsorption of the ions on the grain facets slows down crystal growth [42,43], therefore, higher adsorption of ions on the crystal nuclei results in lower particle size. The ionic radius decreases in the row La^3+^-Sm^3+^-Gd^3+^-Y^3+^-Lu^3+^, therefore, surface charge density increases in this order. Nucleation is faster for ions with larger ionic radius, which means that this process slows down in the row La^3+^-Sm^3+^-Gd^3+^-Y^3+^-Lu^3+^. Adsorption of Cit^3−^ and Na^+^ ions is more pronounced for the particles with higher surface charge density increasing from La^3+^ to Lu^3+^. Therefore, the observed particle size reduction upon substitution of the yttrium ion to La^3+^, Sm^3+^, and Gd^3+^ ions is dominated by the decrease in crystal growth rate due to the adsorption of Na^+^ and Cit^3−^ ions inhibiting crystal growth. We assume that from Gd to Lu, the crystal growth rate changes insignificantly because the large amount of Na^+^ and Cit^3−^ ions covers the crystal grain surface, and additional Na^+^ and Cit^3−^ adsorption is not favorable anymore. At the same time, the nucleation rate monotonically decreases from La^3+^ to Lu^3+^, which explains the particle size growth upon substitution of the yttrium by lutetium ions. We found that co-doping of the large amounts of La^3+^ ions results in the formation of the two types of hexagonal particles of significantly different sizes (Figure 4e,f). Thus, the NaY_0.58_Sm_0.02_La_0.4_F_4_ compound consists of large (1517 ± 64 nm) and small (254 ± 16 nm) particles. The average size of the NaY_0.38_Sm_0.02_La_0.6_F_4_ sample also contains two sorts of particles with an average size of 1916 ± 132 and 102 ± 9 nm. The fraction of the smaller particles significantly increases from 40 to 60 at.% La^3+^, therefore, according to PXRD data, we assume that larger particles correspond to β-NaLnF_4_ and smaller particles are attributed to the LnF_3_ crystalline phase.

### 3.3. Luminescence Properties

**Figure 8:**
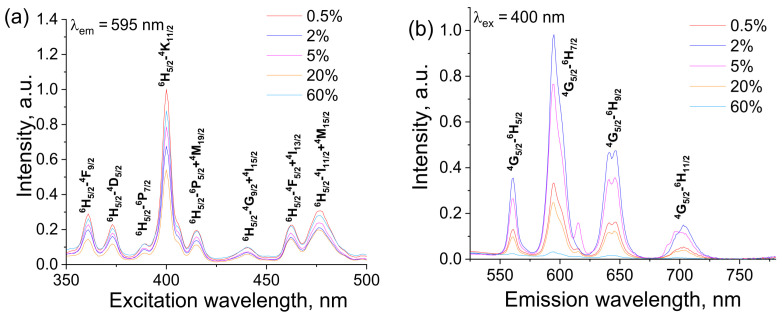

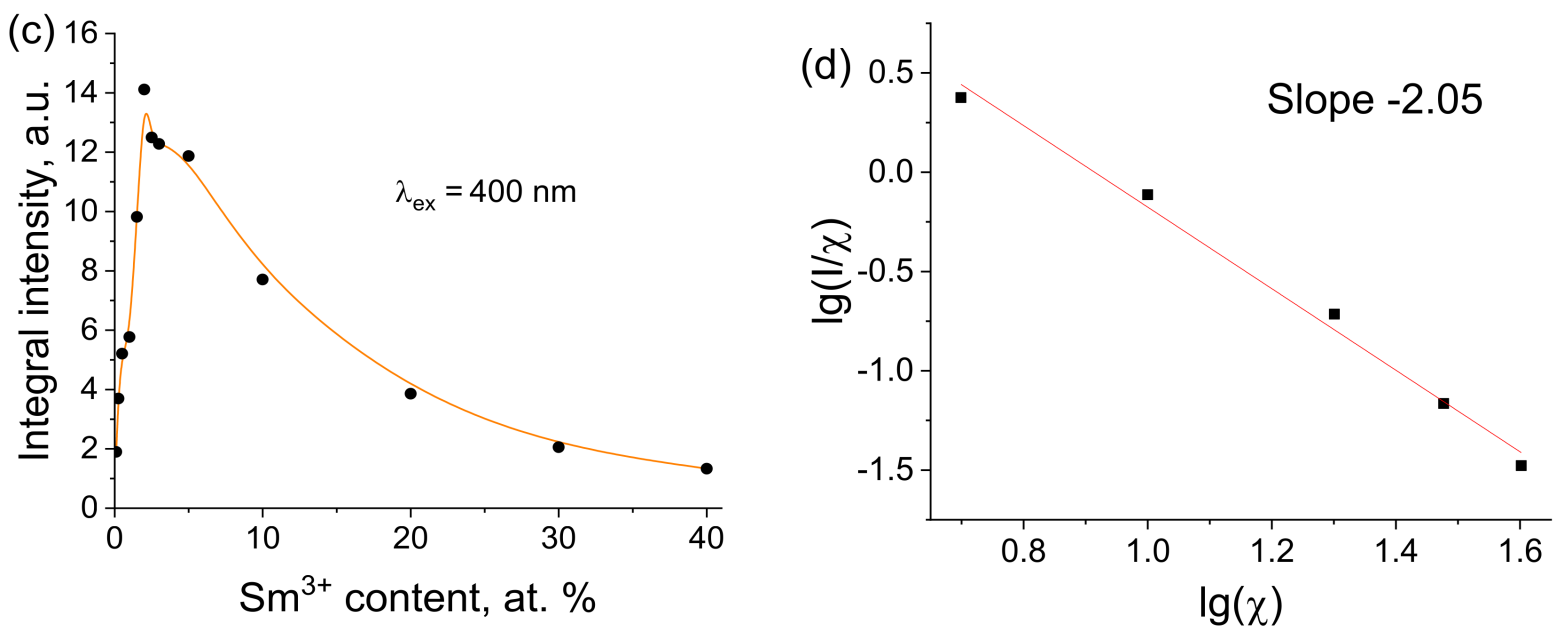
The luminescence excitation (**a**) emission (**b**) spectra of NaY_1-x_Sm_x_F_4_ concentration series; dependence of integral intensities of ^5^G_5/2_ → ^6^H_7/2_ emission band on Sm^3+^ concentration (**c**), logarithmic plot NaY_1−x_Sm_x_F_4_ of emission integral intensity dependence on dopant concentration fitted to the linear function (**d**).

Excitation spectra of NaY_1−x_Sm_x_F_4_ samples monitored at the ^5^G_5/2_ → ^6^H_7/2_ (595 nm) transition were in the spectral range of 350–500 nm, Figure 8a. One can see that spectra consist of sharp peaks attributed to the f-f electron transitions of the Sm^3+^ ion: ^6^H_5/2_ → ^4^F_9/2_ (361 nm), ^6^H_5/2_ → ^4^D_5/2_ (373 nm), ^6^H_5/2_ → ^6^P_7/2_ (389 nm), ^6^H_5/2_ → ^4^K_11/2_ (400 nm), ^6^H_5/2_ → ^6^P_5/2_ + ^4^M_19/2_ (415 nm), ^6^H_5/2_ → ^4^G_9/2_ + ^4^I_15/2_ (440 nm), ^6^H_5/2_ → ^4^F_5/2_ + ^4^I_13/2_ (462 nm) and ^6^H_5/2_ → ^4^I_11/2_ + ^4^M_15/2_ (476 nm). The ^6^H_5/2_ → ^4^K_11/2_ transition centered at 400 nm is dominated in the obtained spectra. Figure 8b presents emission spectra of NaY_1−x_Sm_x_F_4_ concentration series upon 400 nm excitation into the ^6^H_5/2_ → ^4^K_11/2_ band. Emission spectra included lines corresponding to transitions from excited ^4^G_5/2_ to lower ^6^H_J_ levels: ^4^G_5/2_ → ^6^H_5/2_ (561 nm), ^5^G_5/2_ → ^6^H_7/2_ (595 nm), ^4^G_5/2_ → ^6^H_9/2_ (641, 646 nm) and ^4^G_5/2_ → ^6^H_11/2_ (703 nm). The most prominent transition in the spectra was the ^5^G_5/2_ → ^6^H_7/2_ transition. Analysis of the emission spectra has demonstrated that the spectral shape excitation and emission spectra do not depend on the Sm^3+^ content, whereas the Sm^3+^ doping concentration significantly affected the emission intensity, Figure 8a,b. The concentration dependence of integral intensities of the ^5^G_5/2_ → ^6^H_7/2_ emission band is presented in Figure 8c. The emission intensity non-monotonically depends on the Sm^3+^ concentration reaching the maximum at the Sm^3+^ content of 2 at.% (x = 0.02). Such type of concentration dependence can be explained by the two competitive effects in phosphors upon Sm^3+^ concentration rise [44,45]. Thus, the rise of the number of luminescent sites results in radiative emission probability increase and, as a result, the emission intensity increase. At the same time, upon Sm^3+^ concentration rise, the distance between Sm^3+^ ions decreases resulting in the nonradiative processes probability increase, which leads to the emission quenching. If doping ions occupy a single crystallographic position in the host, the energy transfer mechanism is determined by the critical energy transfer distance (R_c_). This distance can be calculated by the following formula [46]:

(1) $$R_{c}=2{\left(\frac{3V}{4{\pi \chi }_{c}N}\right)}^{\frac{1}{3}},$$

where $\chi_{c}$ is a critical concentration of luminescent ion (0.02), V is unit cell volume for NaY_0.98_Sm_0.02_F_4_ (109.64 Å^3^), N—number of cation sites in crystal structure (1.5 for β-NaYF_4_ [47]). Using these parameters, the critical energy transfer distance $R_{c}$ in NaY_1−x_Sm_x_F_4_ is calculated to be of 19.11 Å. According to Blasse theory [46], when $R_{c}$ > 5 Å, the main contribution to non-radiative energy transfer occurs by the multipole–multipole interactions. At high samarium (III) concentration, the probability of radiative emission is constant; therefore, the energy transfer between Sm^3+^ ions in the NaYF_4_ host is dominated by the multipole–multipole interactions. For the determination of interaction type, Van Uitert [48] proposed an equation, which later was modified by Ozawa and Jaffe [49]:

(2) $$\frac{I}{\chi }=\frac{k}{1+{\beta \chi }^{\frac{\theta }{3}}},$$

where I is integral intensity, χ is the concentration of the luminescent ion. Assuming that ${\beta \chi }^{\frac{\theta }{3}}$ ≫ 1, one can build the linearized coordinates $\mathrm{lg}\frac{I}{\chi }-\mathrm{lg}\chi$ (Figure 8d). Linear fitting of dependence in these coordinates gives the value $\frac{\theta }{3}$ = 2.05. It is known that dipole–dipole, dipole–quadrupole, and quadrupole–quadrupole interactions correspond to θ values of 6, 8, and 10, respectively [50]. For NaY_1−x_Sm_x_F_4_, θ = 6, therefore nonradiative energy transfer between samarium (III) ions in the NaYF_4_ host is caused by dipole–dipole interactions.

**Figure 9:**
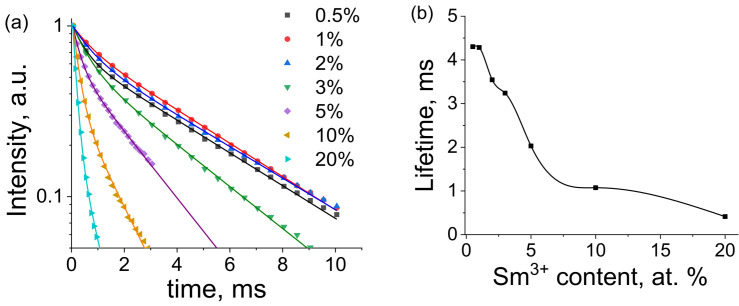
(**a**) Luminescence decay curves of NaY_1−x_Sm_x_F_4_ phosphors monitored at 595 nm upon 400 nm excitation; and (**b**) doping concentration effect on ^5^G_5/2_ level lifetime. Experimental values and best biexponential fits are shown as dots and lines, respectively.

Luminescence decay curves of NaY_1−x_Sm_x_F_4_ phosphors monitored at 595 nm (^5^G_5/2_ → ^6^H_7/2_ transition) upon 400 nm excitation are presented in Figure 9a. All experimental decay curves displayed non-single exponential behavior and, therefore, bi-exponential models were applied for fitting (Equation (3)). The best-fit parameters are given in Table S5 (Supplementary Materials). Bi-exponential decay of small-sized materials is usually explained by the presence of two types of luminescent ions situated in the volume and on the surface of the particles, which have different decay times [51,52]. Sm^3+^ ions situated on the surface display lower lifetimes due to a higher probability of quenching.

(3) $$I\left(t\right)=A_{1}e^{\frac{t}{\tau_{1}}}+A_{2}e^{\frac{t}{\tau_{2}}},$$

where A_1_ and A_2_ are pre-exponential constants, and τ_1_ and τ_2_ are fitting lifetimes.

Average luminescence lifetime (τ_av_), which corresponds to the ^5^G_5/2_ level lifetime, was calculated according to the following equation to simplify comparison [53,54]:

(4) $$\tau_{\mathrm{av}}=\frac{A_{1}\tau_{1}^{2}+A_{2}\tau_{2}^{2}}{A_{1}\tau_{1}+A_{2}\tau_{2}},$$

The Sm^3+^ concentration dependence of the obtained lifetimes is shown in Figure 9b. One can see a monotonic decrease in the lifetimes from 4.3 ms to 0.4 ms along with the increase in samarium concentration. Such behavior is most likely linked to the growth of the nonradiative decay rate due to the increase in spatial energy migration followed by further quenching of impurities.

**Figure 10:**
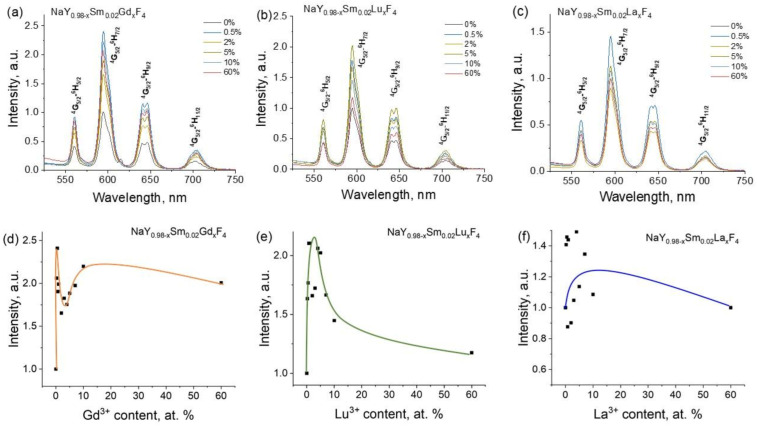
The emission spectra of synthesized compounds NaY_0.98−x_Sm_0.02_Ln_x_F_4_ (Ln = Gd, Lu, La on (**a**–**c**), respectively), upon 400 nm excitation; dependence of integral intensities of ^5^G_5/2_ → ^6^H_7/2_ emission band (595 nm) on Gd^3+^ (**d**), Lu^3+^ (**e**), and La^3+^ (**f**) content) relative to the NaY_0.98_Sm_0.02_F_4_ sample.

**Figure 11:**
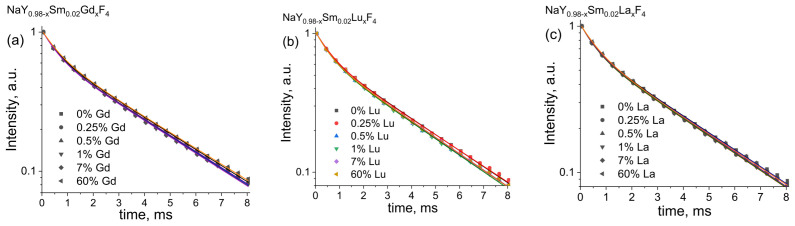
Luminescence decay curves of NaY_0.98−x_Sm_0.02_Ln_x_F_4_ (Ln = Gd, Lu, La on the panels (**a**–**c**), respectively), phosphors monitored at 595 nm upon 400 nm excitation. Experimental values and best biexponential fits are shown as dots and lines, respectively.

Further studies were devoted to the co-doping effect of non-luminescent Gd^3+^, Lu^3+^, and La^3+^ ions on the luminescence properties of NaYF_4_: Sm^3+^ powders. As was demonstrated earlier, Sm^3+^ optimum concentration is 2%, so this samarium concentration was used for samples with Gd^3+^, Lu^3+^, and La^3+^ co-doping. Emission spectra of NaY_0.98−x_Sm_0.02_Ln_x_F_4_ (Ln = Gd, Lu, La) compounds upon 400 nm excitation, Figure 10a–c. One can notice that Gd^3+^, Lu^3+^, and La^3+^ co-doping affect only the emission intensity and alternate neither the positions of the emission bands corresponding to ^4^G_5/2_-^6^H_J_ transitions nor their relative intensities. In order to estimate this effect, the integral emission intensities corresponding to the most intense ^5^G_5/2_ → ^6^H_7/2_ transition of Sm^3+^ ions (595 nm) were calculated and plotted in Figure 10d–f relative to the NaY_0.98_Sm_0.02_F_4_ sample. We found that co-doping by the abovementioned rare earth ions results in an increase in the luminescence intensities. Thus, the substitution of Y^3+^ ion by Gd^3+^ results in the most emission enchantment up to 2.4 times, Figure 10d. The maximum emissions intensities are observed for the Gd^3+^ content of 0.5 and 10 at.% corresponding to the increase in the luminescence intensity at 2.4, and 2.2 times, respectively. The co-doping of NaY_0.98_Sm_0.02_F_4_ compound by Lu^3+^ ion results in emission enchantment up to 2.1 times, the maximum effect is observed for the lutetium content of 1 at.%, Figure 10e. The least prominent effect is observed for co-doping of NaY_0.98_Sm_0.02_F_4_ materials by La^3+^ ion, where the emission enchantment is barely noticeable, Figure 10f. Therefore, it is difficult to mention the precise position of the La^3+^ concentration corresponding to the largest effect. To reveal the mechanism of the luminescence enhancement effect by Gd^3+^, Lu^3+^, and La^3+^ co-doping, the luminescence kinetics was studied for the samples with various concentrations of co-doping ions. Luminescence decay curves of NaY_0.98−x_Sm_0.02_Ln_x_F_4_ (Ln = Gd, Lu, La) phosphors monitored at 595 nm (^5^G_5/2_ → ^6^H_7/2_ transition) upon 400 nm excitation are presented in Figure 11. All experimental decay curves displayed non-single exponential behavior and two exponential models were applied for fitting (Equation (3)). The best-fit parameters are given in Tables S6–S8 (Supplementary Materials). The average luminescence lifetimes, which correspond to the ^5^G_5/2_ level lifetimes, were calculated using Equation (4) and given in Table 1. We revealed that co-doping of NaY_0.98_Sm_0.02_F_4_ by Gd^3+^, Lu^3+^, and La^3+^ does not result in a change in the ^5^G_5/2_ excited state lifetime. Therefore, the substitution of yttrium ions by gadolinium, lutetium, and lanthanum ions does not change the probability of the ^4^G_5/2_-^6^H_J_ radiative transition.

Emission enhancement resulting from Gd^3+^, Lu^3+^, and La^3+^ co-doping of NaY_0.98_Sm_0.02_F_4_ materials, in principle, can be caused by the absorption and/or emission probability increase. However, in the second case, excited state lifetimes must change, which is not observed in our experiments. Therefore, one can conclude, that doping by Ln^3+^ ions results in changing only extinction coefficients due to the changing probability of symmetry forbidden ^6^H_5/2_ → ^4^K_11/2_ transition. Luminescence intensity enhancement resulted from co-doping of Eu^3+^-containing materials by non-luminescent ions such as Bi^3+^, Gd^3+^, alkali, and alkali earth metal ions was reported previously [11,26,27,55,56,57,58,59,60,61,62,63,64]. The observed effect was explained by structure distortion due to the difference between radii of substituted and doping ions resulting in the increase in the emission and absorption probabilities. In our case, Gd^3+^, Lu^3+^, and La^3+^ co-doping of NaY_0.98_Sm_0.02_F_4_ materials at low concentrations of the dopant results in symmetry lowering of Sm^3+^ local environment that leads to an increase in the absorption probability and, obviously, extinction coefficients [57,65]. Indeed, the maximum emission effect is observed when about 1% yttrium ions are substituted with gadolinium or lutetium ions. Meanwhile, compounds containing a significant amount of gadolinium ions also demonstrate larger emission intensity than NaY_0.98_Sm_0.02_F_4_. A similar effect was observed by Martins and co-workers [66] where co-doping of Y_2_O_3_: Eu^3+^ by Gd^3+^ ions resulted in an increase in the emission intensity. They explain this phenomenon of partial absorption by the host Gd_2_O_3_ matrix followed by the energy transfer to Gd^3+^ ion, and then from Gd^3+^ to Eu^3+^ ion. It is known, that the β-NaYF_4_ host absorbed light at 200–450 nm [7]. Most probably, the addition of Gd^3+^ ion results in the more prominent absorption of β-NaYF_4_: Gd^3+^ matrix at the same range of UV spectrum. We propose that 400 nm excitation of NaY_0.98−x_Sm_0.02_Gd_x_F_4_ promotes β-NaYF_4_: Gd^3+^ host matrix into the excited state (in parallel with ^6^H_5/2_ → ^4^K_11/2_ transition of Sm^3+^ ion) followed by energy transfer from the host matrix to Sm^3+^ ions, which results in increases in luminescence intensities relative to NaY_0.98_Sm_0.02_F_4_.

## 4. Conclusions

In the present work, four series of NaYF_4_ particles doped with Sm^3+^, Gd^3+^, Lu^3+^, and La^3+^ ions, NaY_1−x_Sm_x_F_4_ (x = 0–0.5) and NaY_0.98−y_Sm_0.02_Ln_y_F_4_ (Ln = Gd, Lu, La; y = 0–0.6), were synthesized by a hydrothermal method at a temperature of 180 °C using citric acid as a stabilizing agent. Analysis of PXRD patterns demonstrated that NaY_1−x_Sm_x_F_4_ and NaY_0.98−x_Sm_0.02_Ln_x_F_4_ (Ln = Lu, Gd) have similar crystal structures corresponding to the hexagonal β-NaYF_4_. For the NaY_0.98−x_Sm_0.2_La_x_F_4_ series, the β-NaYF_4_ crystalline phase is dominated at La^3+^ content up to 20%. At higher La^3+^ concentrations, the solid solutions are formed as a LaF_3_ crystalline phase. Among the β-NaYF_4_ phase, unit cell volumes linearly depend on dopant concentration, which demonstrates that Sm^3+^, Gd^3+^, Lu^3+^, and La^3+^ ions isomorphically substitute Y^3+^ ions in the β-NaYF_4_ structure. Sm^3+^, Gd^3+^, and La^3+^ doping results in unit cell volumes increase because the Y^3+^ ion has a smaller ionic radius (1.075 Å) than Sm^3+^ (1.132 Å) and Gd^3+^ (1.107 Å) ions. The substitution of Y^3+^ ions by smaller Lu^3+^ ions (1.032 Å) leads to unit cell volume reduction. According to SEM data, particles of all synthesized compounds have the shape of hexagonal prisms and sizes ranging from 46 to 1916 nm depending on the sample composition. In the NaY_1−x_Sm_x_F_4_ series, the substitution of Y^3+^ by Sm^3+^ ions leads to the particle size reduction from 682 nm (NaYF_4_) down to 78 nm (NaY_0.5_Sm_0.5_F_4_). Co-doping of NaY_0.98_Sm_0.02_F_4_ by La^3+^ and Lu^3+^ ions results in particle size increases due to faster growth (for La^3+^) and slower nucleation (for Lu^3+^) [10]. In contrast to La^3+^ and Lu^3+^, co-doping of these materials by Gd^3+^ ions leads to particle size reduction because the lowest growth/nucleation rates are characteristic of Gd^3+^. All synthesized compounds demonstrate photoluminescence under 400 nm excitation (^6^H_5/2_ → ^4^K_11/2_ transition in Sm^3+^). Experimental Sm^3+^ optimal doping concentration in β-NaYF_4_ host is 2%. Further increasing of Sm^3+^ concentration leads to strong quenching due to dipole–dipole interactions between Sm^3+^ ions. We demonstrated that co-doping by different non-luminescent Ln^3+^ ions (where Ln is not only Gd, but also La and Lu) in low dopant concentration (in the range from 0 to 10 at.%) results in increasing luminescent intensities. Co-doping of NaY_0.98_Sm_0.02_F_4_ by Gd^3+^, Lu^3+^, and La^3+^ ions does not lead to the change in ^4^G_5/2_ excited state lifetimes, therefore co-doping by non-luminescent ions leads to increase in absorption probability due to the Sm^3+^ local symmetry distortion. Therefore, we discovered that enhancement of luminescence intensity as a result of co-doping by non-luminescent Gd^3+^, Lu^3+^, and La^3+^ ions is a general phenomenon and can be applied to improve the optical properties of a wide range of inorganic REE-containing phosphors.

## Figures and Tables

**Table 1:** Lifetimes of ^4^G_5/2_ excitation state of Sm^3+^ ion in NaY_0.98-x_Sm_0.02_Ln_x_F_4_ (Ln = Gd, Lu, La).

Ln^3+^Content, at.%	Ln^3+^ = Gd^3+^	Ln^3+^ = Lu^3+^	Ln^3+^ = La^3+^
	τ_av_, ms	τ_av_, ms	τ_av_, ms
0	3.54 ± 0.05	3.54 ± 0.05	3.54 ± 0.05
0.25	3.51 ± 0.05	3.58 ± 0.05	3.46 ± 0.05
0.5	3.50 ± 0.05	3.46 ± 0.05	3.55 ± 0.05
1	3.47 ± 0.05	3.42 ± 0.05	3.46 ± 0.05
7	3.46 ± 0.05	3.46 ± 0.05	3.47 ± 0.05
60	3.58 ± 0.05	3.45 ± 0.05	3.53 ± 0.05

## Data Availability

The data presented in this study are available in this article.

## Supplementary Materials

The following supporting information can be downloaded at: https://www.mdpi.com/article/10.3390/ma16062157/s1, Table S1: Unit cell parameters of the NaY_(1−x)_Sm_x_F_4_ samples; Table S2: Unit cell parameters of the NaY_(0.98−x)_Sm_0.02_La_x_F_4_ samples; Table S3: Unit cell parameters of the NaY_(0.98−x)_Sm_0.02_Gd_x_F_4_ samples; Table S4: Unit cell parameters of the NaY_(0.98−x)_Sm_0.02_Lu_x_F_4_ samples; Table S5: Pre-exponential constants, fitting lifetimes, and average luminescence lifetimes of NaY_(1−x)_Sm_x_F_4_ powders; Table S6: Pre-exponential constants, fitting lifetimes and average luminescence lifetimes of NaY_(0.98−x)_Sm_0.02_Gd_x_F_4_ powders; Table S7: Pre-exponential constants, fitting lifetimes and average luminescence lifetimes of NaY_(0.98−x)_Sm_0.02_Lu_x_F_4_ powders; Table S8: Pre-exponential constants, fitting lifetimes and average luminescence lifetimes of NaY_(0.98−x)_Sm_0.02_La_x_F_4_ powders.
