# Supplementary material for: Effect of Gd3+, La3+, Lu3+ Co-Doping on the Morphology and Luminescent Properties of NaYF4:Sm3+ Phosphors
Source: Materials (Basel). 2023 Mar 7;16(6):2157. doi: 10.3390/ma16062157 (PMC10058261; doi:10.3390/ma16062157)
Supplement: Supplementary file 1 [file materials-16-02157-s001.zip › materials-2245672-supplementary.pdf]

materials

Supplementary Materials

Effect of Gd^{3+}, La^{3+}, Lu^{3+} Co-Doping on the Morphology and Luminescent Properties of $NaYF_4$:Sm^{3+} Phosphors

Viktor G. Nosov [1], Anna A. Betina [1], Tatyana S. Bulatova [1], Polina B. Guseva [1], Ilya E. Kolesnikov [1], Sergey N. Orlov [1,2,3], Maxim S. Panov [1,4], Mikhail N. Ryazantsev [1,5], Nikita A. Bogachev [1], Mikhail Yu Skripkin [1,*] and Andrey S. Mereshchenko [1,*]

[1] Saint-Petersburg State University, 7/9 Universitetskaya Emb., 199034 St. Petersburg, Russia
[2] Federal State Unitary Enterprise "Alexandrov Research Institute of Technology", 72 Koporskoe Shosse, 188540 Sosnovy Bor, Russia
[3] Institute of Nuclear Industry, Peter the Great St. Petersburg Polytechnic University (SPbSU), 29, Polytechnicheskaya Street, 195251 St. Petersburg, Russia
[4] Center for Biophysical Studies, Saint Petersburg State Chemical Pharmaceutical University, 14 Professor Popov Str., Lit. A, 197022 St. Petersburg, Russia
[5] Nanotechnology Research and Education Centre RAS, Saint Petersburg Academic University, 8/3 Khlopina Street, 194021 St. Petersburg, Russia
* Correspondence: skripkin1965@yandex.ru (M.Y.S.); a.mereshchenko@spbu.ru (A.S.M.); Tel.: +7-906-246-96-32 (M.Y.S.); +7-951-677-54-65 (A.S.M.)

Citation: Nosov, V.G.; Betina, A.A.; Bulatova, T.S.; Guseva, P.B.; Kolesnikov, I.E.; Orlov, S.N.; Panov, M.S.; Ryazantsev, M.N.; Bogachev, N.A.; Skripkin, M.Y.; et al. Effect of Gd^{3+}, La^{3+}, Lu^{3+} Co-Doping on the Morphology and Luminescent Properties of $NaYF_4$:Sm^{3+} Phosphors. *Materials* **2023**, *16*, 2157. https://doi.org/10.3390/ma16062157

Academic Editor: Valery V. Tuchin

Received: 11 February 2023
Revised: 3 March 2023
Accepted: 6 March 2023
Published: 7 March 2023

Copyright: © 2023 by the authors. Submitted for possible open access publication under the terms and conditions of the Creative Commons Attribution (CC BY) license (https://creativecommons.org/licenses/by/4.0/).

The supplementary materials contain unit cell parameters, refined from XRD patterns, pre-exponential constants, fitting lifetimes and average luminescence lifetimes of the synthesized samples.

Table S1. Unit cell parameters of the $NaY_{(1-x)}Sm_xF_4$ samples.

X (Sm^{3+})	a, Å	c, Å	V, Å^3
0	5.9900(5)	3.5268(3)	109.589(19)
0.02	5.9904(6)	3.5281(4)	109.64(2)
0.05	5.9939(5)	3.5325(3)	109.91(2)
0.1	5.9973(5)	3.5368(3)	110.17(2)
0.2	6.0048(5)	3.5469(3)	110.76(2)
0.3	6.0138(4)	3.5583(3)	111.447(17)
0.4	6.0233(4)	3.5704(3)	112.178(18)
0.5	6.0326(4)	3.5839(3)	112.953(19)

Table S2. Unit cell parameters of the $NaY_{(0.98-x)}Sm_{0.02}La_xF_4$ samples.

X (La^{3+})	a, Å	c, Å	V, Å^3
0.005	5.9911(5)	3.5310(3)	109.756(19)
0.02	5.9955(4)	3.5352(2)	110.050(17)
0.05	6.00170(16)	3.54401(19)	110.554(8)
0.1	6.0128(5)	3.5604(3)	111.47(2)
0.2	6.0340(5)	3.5917(3)	113.25(2)
0.4			
0.6			

Table S3. Unit cell parameters of the $NaY_{(0.98-x)}Sm_{0.02}Gd_xF_4$ samples.

X (Gd^{3+})	a, Å	c, Å	V, Å^3
0.005	5.9917(4)	3.5290(3)	109.719(17)
0.02	5.9914(4)	3.5290(3)	109.708(18)
0.05	5.9942(5)	3.5320(3)	109.902(19)
0.1	5.9949(5)	3.5360(3)	110.05(2)
0.2	6.0008(5)	3.5424(3)	110.47(2)
0.4	6.0117(4)	3.5571(2)	111.333(17)
0.6	6.0240(4)	3.5734(2)	112.299(16)

Table S4. Unit cell parameters of the $NaY_{(0.98-x)}Sm_{0.02}Lu_xF_4$ samples.

X (Lu^{3+})	a, Å	c, Å	V, Å^3
0.005	5.9944(3)	3.52989(18)	109.847(11)
0.02	5.9951(3)	3.52818(17)	109.819(11)
0.05	5.9966(4)	3.5296(3)	109.918(17)
0.1	5.9862(7)	3.5219(4)	109.30(3)
0.2	5.98301(15)	3.51629(10)	109.007(6)
0.4	5.97266(10)	3.50358(6)	108.237(4)
0.6	5.95889(9)	3.48396(5)	107.136(4)

Table S5. Pre-exponential constants, fitting lifetimes and average luminescence lifetimes of $NaY_{(1-x)}Sm_xF_4$ powders.

X (Sm^{3+})	t_1, ms	A_1	t_2, ms	A_2	t_{av}, ms
0.005	0.49585	0.36133	4.52447	0.67952	4.30263
0.01	0.69825	0.23256	4.44964	0.79106	4.28421
0.02	0.58935	0.32768	3.7595	0.70596	3.54448
0.03	0.60009	0.41627	3.538	0.62408	3.23940
0.05	0.30837	0.49036	2.2279	0.59149	2.03031
0.1	0.24629	0.78663	1.3877	0.36752	1.07347
0.2	0.13878	1.10607	0.67055	0.24386	0.41307

Table S6. Pre-exponential constants, fitting lifetimes and average luminescence lifetimes of $NaY_{(0.98-x)}Sm_{0.02}Gd_xF_4$ powders.

**X (Gd^{3+})**	**t_1, ms**	**A_1**	**t_2, ms**	**A_2**	**t_{av}, ms**
0	0.58935	0.32768	3.7595	0.70596	3.54448
0.0025	0.58007	0.34455	3.73643	0.69077	3.50958
0.005	0.60648	0.34961	3.74336	0.68448	3.50362
0.01	0.61335	0.35128	3.71315	0.68324	3.47050
0.07	0.60558	0.35478	3.70736	0.67945	3.46359
0.6	0.65792	0.32904	3.81826	0.70232	3.58219

Table S7. Pre-exponential constants, fitting lifetimes and average luminescence lifetimes of $NaY_{(0.98-x)}Sm_{0.02}Lu_xF_4$ powders.

**X (Lu^{3+})**	**t_1, ms**	**A_1**	**t_2, ms**	**A_2**	**t_{av}, ms**
0	0.58935	0.32768	3.7595	0.70596	3.54448
0.0025	0.65557	0.3448	3.82723	0.68725	3.57623
0.005	0.61999	0.36009	3.71785	0.67513	3.46482
0.01	0.59521	0.3532	3.6604	0.68179	3.42225
0.07	0.58935	0.34112	3.68533	0.69298	3.45940
0.6	0.58524	0.34268	3.67685	0.69221	3.45103

Table S8. Pre-exponential constants, fitting lifetimes and average luminescence lifetimes of $NaY_{(0.98-x)}Sm_{0.02}La_xF_4$ powders.

**X (La^{3+})**	**t_1, ms**	**A_1**	**t_2, ms**	**A_2**	**t_{av}, ms**
0	0.58935	0.32768	3.7595	0.70596	3.54448
0.0025	0.58503	0.34648	3.68518	0.68894	3.45597
0.005	0.60215	0.33958	3.78063	0.69506	3.55115
0.01	0.5846	0.34412	3.68805	0.69116	3.46104
0.07	0.59006	0.3325	3.69152	0.70171	3.47315
0.6	0.6829	0.3456	3.78738	0.68578	3.52878
